# Mice Undergo Organ-Specific Transcriptional and Metabolic Remodeling During Late Adolescence

**DOI:** 10.21203/rs.3.rs-10171816/v1

**Published:** 2026-08-05

**Authors:** Qing-xia Huang, Ahmad Al Ajami, Eva Kohnert, Temel Kilic, Christina Engel, Melanie Boerries, Clemens Kreutz, Katharina Moos, Gerd Walz

**Affiliations:** University Medical Center Freiburg; University Medical Center and Faculty of Medicine; University Medical Center Freiburg; University Medical Center Freiburg; University Medical Center Freiburg; University Medical Center and Faculty of Medicine; University Medical Center Freiburg; University Medical Center and Faculty of Medicine; University Medical Center Freiburg

**Keywords:** Organ-specific maturation, transcriptional changes, metabolic reprogramming, lipid metabolism, gut-liver axis

## Abstract

Mouse models aimed at deciphering human aging have typically focused on early development or late-life stages. Much less is known about the transition from late adolescence to young adulthood, spanning 8 to 12 weeks of age. Here, we show that C57Bl6 mice undergo a comprehensive developmental transition characterized by significant transcriptional and metabolic reprogramming during this time. Transcriptional analysis revealed substantial, organ-specific changes: the heart shifts toward a maintenance-focused phenotype with down-regulated oxidative phosphorylation (OXPHOS) and increased expression of genes supporting structural remodeling. Conversely, the kidney adopts an immunologically active state, reflecting progressive immune cell colonization, while the liver shifts from a hematopoietic role to one dominated by lipid metabolism, detoxification, and ketogenesis. These transcriptional changes correspond to a coherent metabolic maturation driven by the gut-liver axis. Intestinal maturation enhances lipid absorption and microbial fermentation, leading to increased circulating levels of short-chain fatty acids, poly-unsaturated glycerolipids, and sphingomyelins. Elevated hepatic production of ketone bodies, driven by up-regulated fatty acid metabolism genes, provides an efficient alternative energy substrate for peripheral tissues like the heart. Urinary metabolite profiles, marked by increased acylglycines and tricarboxylic acid (TCA) cycle intermediates, further confirm heightened mitochondrial energy turnover and fatty acid β-oxidation. Collectively, this coordinated maturation from 8 to 12 weeks signifies the transition from a highly plastic developmental state to a stable, energy-efficient, and metabolically flexible adult phenotype.

## Introduction

Between 8 and 12 weeks of age, a period that largely corresponds to a human lifespan between 10 and 15 years [1], mice transition from late adolescence into young adulthood, reaching sexual maturity and physiological stabilization in most organ systems. At this stage mice have completed rapid postnatal growth, but not yet reached adulthood and age-related decline. During this age range, developmental processes are ongoing, resulting in increased bone mass, T- and B-lymphocyte production and brain size [2]. In addition, there is also a significant change in behavioral patterns, including burrowing, nest building and voluntary wheel running [3].

The maturation of the postnatal mouse heart is governed by a tightly coordinated series of cellular, molecular, and structural transformations [4, 5]. These changes convert the fetal heart, initially reliant on proliferative cardiomyocytes, into a mature organ. Shortly after birth, cardiomyocytes briefly retain limited proliferative activity to expand heart mass, but this capacity diminishes rapidly as cells become predominantly post-mitotic [6]. Concurrently, the heart transitions from fetal to adult sarcomeric protein isoforms, while development of gap junctions and ion channels supports synchronized cardiac conduction and contraction. A metabolic shift marks this maturation: fetal hearts mainly depend on glycolysis, but as development proceeds, fatty acid oxidation becomes the principal energy source. By postnatal day 7, oxidative metabolism predominates, accompanied by robust mitochondrial biogenesis [7], and a rapid reduction in the heart’s regenerative potential [8]. By approximately day 21, sarcoplasmic reticulum calcium handling and myofibrillar functions mature to adult levels [4].

Although fetal and early postnatal heart development have been extensively characterized, the molecular and metabolic programs that govern adolescent cardiac maturation remain poorly defined. To address this gap, we profiled transcriptional changes in mouse hearts at 8, 10, and 12 weeks of age and compared them with transcriptional changes in the kidney and liver. In addition, we analyzed intestinal, plasma, and urine metabolites, together with the intestinal microbiome, to determine whether organ-specific transcriptional shifts are linked to systemic metabolic remodeling. Our analysis revealed a surprising decline in oxidative phosphorylation capacity during late adolescent cardiac development. This suggests that, rather than simply increasing mitochondrial output, the heart undergoes further refinement as it approaches functional maturity. Collectively, our findings indicate that late adolescence to early adulthood is a period of coordinated systemic remodeling in which the gut, liver, and heart operate as an integrated network to support the transition into a mature organism.

## Results & Discussion

### Transcriptional changes between week 8 and 12 highlight ongoing maturation and adaptation

Between 8 and 12 weeks of age, male C57BL/6 mice exhibited a significant increase in body weight (**Suppl. Figure 1**). Notably, this weight gain occurred without a parallel increase in food or water intake, suggesting a fundamental shift in the efficiency of nutrient processing and energy metabolism.

**Figure 1:**
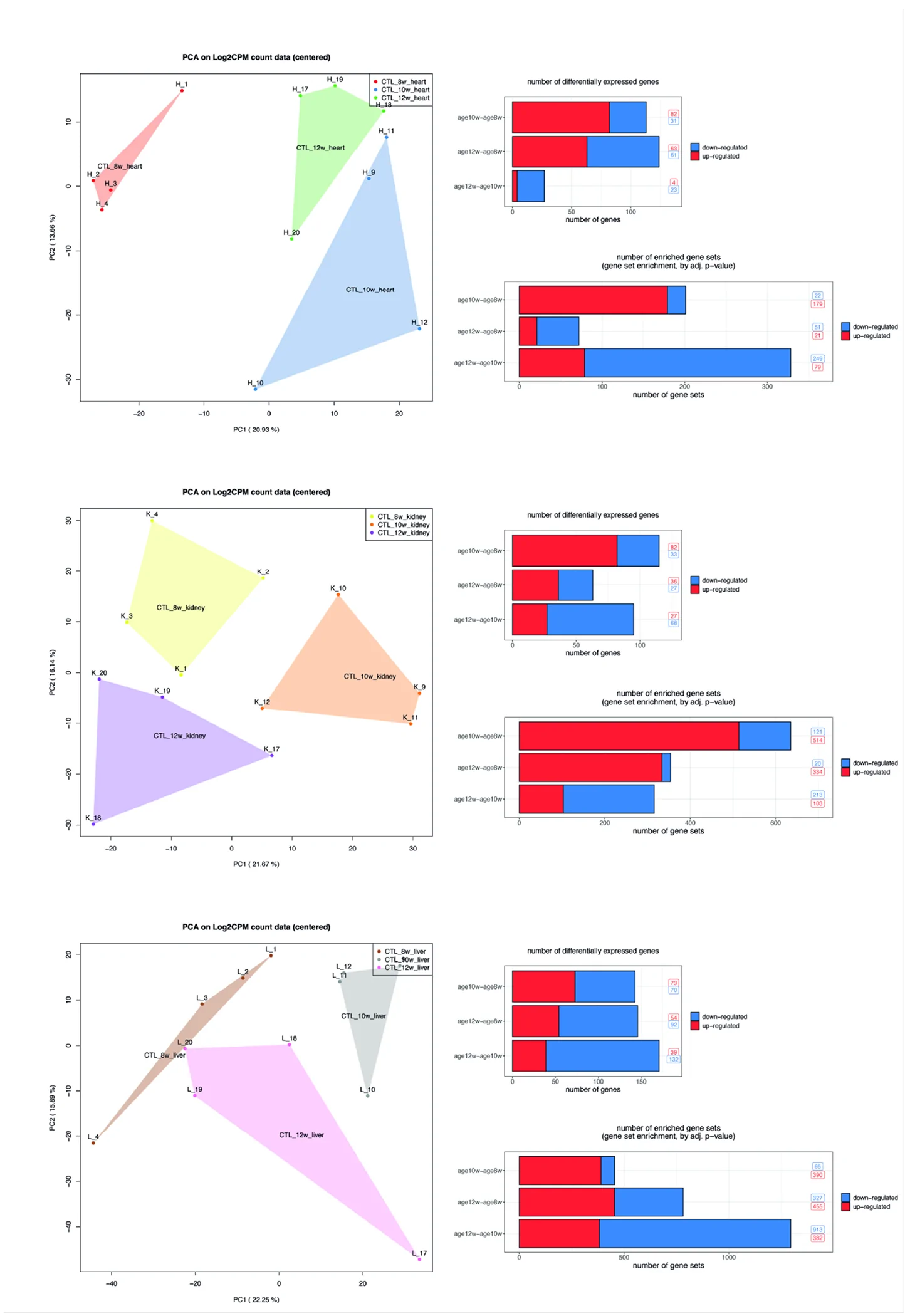
Global transcriptional reorganization during late adolescence. Principal component analysis (PCA) demonstrates distinct temporal separation of transcriptional profiles across the heart, kidney, and liver between 8 and 12 weeks of age. Differential expression analysis between these time points reveals significant gene regulation: heart (63/124 genes; 49% up-regulated), kidney (36/63; 43% up-regulated), and liver (54/146; 63% up-regulated).

Principal component analysis (PCA) of RNA sequencing data revealed distinct clustering of samples from the three time points, demonstrating pronounced transcriptional changes in all three organs between 8 and 12 weeks of age (Fig. 1). Comparison between week 8 and 12 showed that 63 (61) genes were up-(down-) regulated in the heart, 36 (27) genes in the kidney, and 54 (92) genes in the liver (adjusted p-value < 0.05, log2FC |> 1|), suggesting a substantial shift in developmental programs during the relatively short time window of four weeks (Fig. 1).

Gene Set Enrichment Analysis (GSEA) revealed an unexpected down-regulation of pathways involved oxidative phosphorylation in the heart, when mice at 12 weeks of age were compared to 8-week-old mice (**Suppl. Figure 2A, Suppl. Table 1**). In contrast, GSEA terms associated with sarcomeric and contractile maturation were increased, highlighting the ongoing differentiation of cardiomyocytes.

A striking activation of pathways associated with immune functions was detected in 12-week-old kidneys (**Suppl. Figure 2B, Suppl. Table 2**), indicating that the mouse kidney is progressively colonized by diverse immune cell populations in late adolescence. In contrast to the heart, components of the oxidative pathway were increased, signaling the demand for energy to support the active transport processes in the kidney.

Rac signaling is required for renal morphogenesis, which ends in mice around postnatal day 3. Continued Rac signaling past postnatal day 3 supports papillary nephron elongation and nephron differentiation, while down-regulation of Rac signal transduction at week 12 (NES – 1.82; adjusted p-value 0.028) (**Suppl. Table 2**) indicates that the kidney has reached its mature capacity [9, 10].

The top GSEA term revealed up-regulation of fatty acid metabolism in the liver, while down-regulation of genes involved in collagen and extracellular matrix dynamics indicated that the structural maturation of the liver is largely completed in mice by 12 weeks of age (**Suppl. Figure 2C, Suppl. Table 3**).

### Switch to a maintenance-focused cardiac phenotype

**Figure 2:**
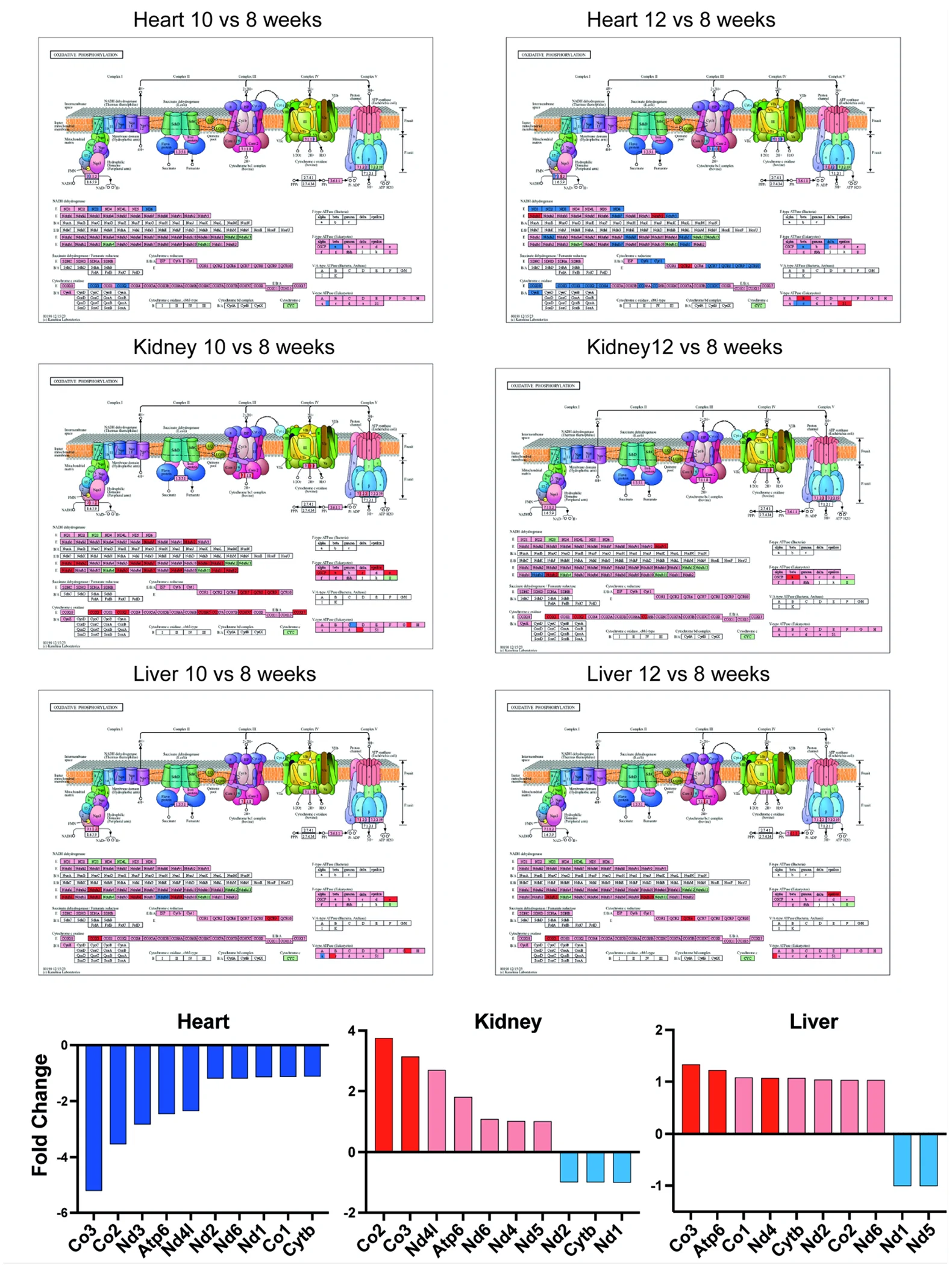
Divergent regulation of the mitochondrial oxidative phosphorylation (OXPHOS) pathway across organs during late adolescence. **(a)** KEGG pathway maps showing expression changes in mitochondrial respiratory chain components (10 and 12 weeks vs. 8 weeks). The heart exhibits a prominent down-regulation of respiratory chain genes, while the kidney and liver show compensatory increases in expression. The map was generated using KEGG Mapper with kind permission of the Kaneshisa Laboratories (https://www.kegg.jp/kegg/kegg1.html). **(b)** Quantified expression changes of key mitochondrial genes (*Co2, Co3, Nd3, Atp6, Nd4l, Nd6, Nd1, Cytb*) at 12 weeks compared to 8 weeks, highlighting the systematic down-regulation in the heart versus the up-regulation observed in the liver and kidney. Red: significantly upregulated genes (adjusted p-value < 0.05), blue: significantly downregulated genes (adjusted p-value < 0.05), pink: genes that were measured but are not significant, and green: genes not present in the dataset.

Several genes of the mitochondrial respiratory chain were significantly downregulated in the heart; in contrast, both kidney and liver displayed increased expression of mitochondrial components to support oxidative phosphorylation (Fig. 2A,B). In 10-week-old mice, the heart has largely completed hypertrophic growth, switching to a fine-tuned metabolic program. Mitochondrial genes (e.g. *mt-Co2*, *mt-Co3*, *mt-Nd3*) decrease as the heart stabilizes its energy usage and moves away from a state requiring rapid remodeling and cellular proliferation. In addition, down-regulation of oxidative phosphorylation (OXPHOS) can serve protective purposes, limiting OXPHOS-associated reactive oxygen species (ROS) production. Although ROS play essential roles in cardiomyocyte differentiation [11], it may be important to curtail oxidative stress during late adolescence.

**Figure 3:**
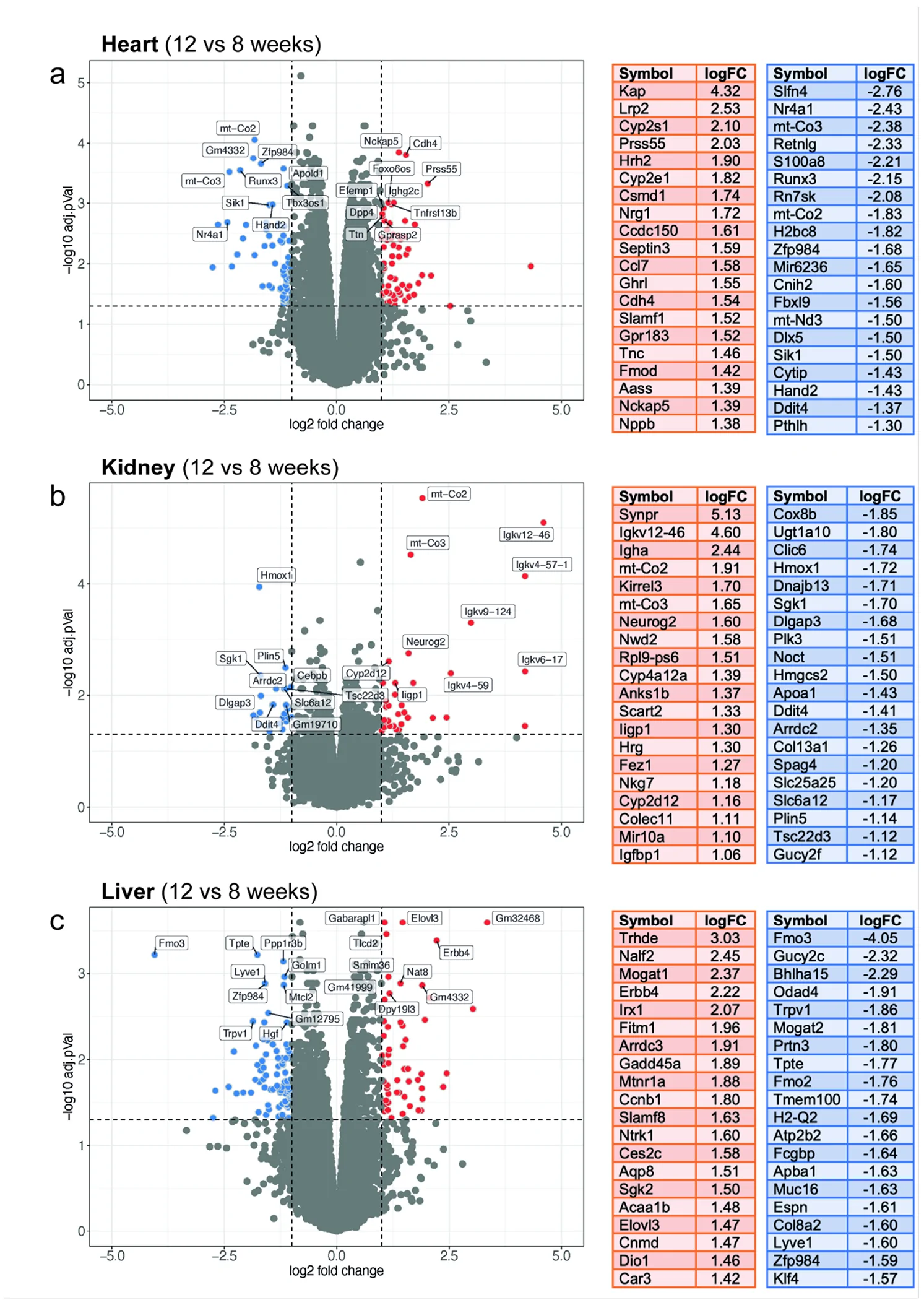
Organ-specific transcriptional profiling reveals distinct maturation signatures in the heart, kidney, and liver at 12 weeks compared to 8 weeks. Volcano plots illustrate differentially expressed genes (DEGs) with corresponding top up-regulated (orange) and down-regulated (blue) gene lists for the **(a)**heart, (**b)** kidney, and **(c)** liver. Analysis highlights unique adaptive responses: the heart prioritizes structural and matrix remodeling genes, the kidney shows enrichment in immune-related signaling, and the liver exhibits a robust shift toward metabolic reprogramming and detoxification. Red and blue dots denote significantly up-regulated and down-regulated genes, respectively (p-adj < 0.05).

Analysis of the top up-regulated genes in 12-week-old mice revealed a coordinated response to support myocardial structure and extracellular matrix remodeling. Genes like *Tnc* (Tenacscin C), *Fmod* (Fibromodulin), *Cdh4* (Cadherin 4) and *Nckap5* (NCK Associated Protein 5) sustain cardiac tissue adaptation to mechanical and hemodynamic demands in late adolescence. Upregulation of *Nppb* (Natriuretic Peptide), *Hrh2* (Histamine Receptor), *Ghrl* (Ghrelin), immune-related genes (e.g. *Ccl7*, *Slamf1*, *Gpr183*), and genes involved in metabolic reprogramming such *Nrg1* (Neuregulin), *Fgfr2* (FGF Receptor), and metabolic enzymes such as *Aass* (Aminoadipate-Semialdehyde Synthase), *Cyp2s1/Cyp2e1* (Cytochrome P450 Family 2 Subfamily S Member 1/Subfamily E Member 1) increase metabolic flexibility and hormonal responsiveness, allowing the heart to respond to changes in volume, pressure and inflammation as the animal transitions to adulthood. Down-regulation of *Slfn4* (Schlafen 4), *S100a8* (S100 calcium binding protein A8), and *Retnlg* (Resistin-like gamma) signal a restriction of inflammatory cell recruitment and the resolution of developmental immune activation, protecting against unnecessary tissue injury (Fig. 3A, **Suppl. Table 4**). Small RNA and chromatin regulators (*Rn7sk*, *Mir6236*, *Zfp984*) are also decreased, aligning with the transition from a highly plastic to a more stable, differentiated state.

### Transition of the kidney to an immunological active organ in late adolescence

Gene expression in the kidney of 12-week-old mice was dominated by immune and antibody signaling, reflecting extensive lymphocyte homing (Fig. 3B, Suppl. Table 5). Genes such as *Synpr*, *Neurog2* and *Ptpn5* are involved in synaptic signaling, pointing towards local neural-renal activity. Genes involved in oxidative phosphorylation were not strikingly altered, suggesting a transition from heightened energy demands in the early postnatal period to energy homeostasis and decreased demand for metabolic plasticity. Several genes, including *Sult2a3, Cidea, Lipo2, Acsbg1, Aldh1a3, Cyp4a12a*, orchestrate fat, lipid and steroid metabolism as well as xenobiotic detoxification [12]. Down-regulation of genes involved in stress responses, e.g. *Hmox1* (Heme Oxygenase 1) and circadian regulators such as *Ddit4, Plk3, Cry1/Noct* implies resolution of acute cellular stress and establishment of a mature, stable renal function (Fig. 3B). Overall, the down-regulated genes mark a shutdown of proliferation and stress responses active in early development, and an entry into tissue maintenance and homeostasis.

### Transition to hepatic lipid metabolism and ketogenesis

GSEA analysis in combination with the top regulated genes suggests a global shift from the fetal and early postnatal stage, where the liver functions primarily as an immune and hematopoietic organ, to lipid metabolism and detoxification (Fig. 3C, **Suppl. Figure 2D, Suppl. Table 6**) [13]. While *Mogat1* (Monoacylglycerol-O-acyltrasnferase-1) and *Acaa1b* (Acetyl-Coenzyme A Acyltransferase 1B) are central for triglyceride synthesis and fatty acid β-oxidation, *Elovl3* (Elongation Of Very Long Chain Fatty Acids 3) and *Fitm1* (Fat Storage-Inducing Transmembrane Protein 1) support elongation of fatty acids and lipid droplet formation, ensuring efficient energy storage and mobilization. *Car3* (carbonic anhydrase 3) may be required to maintain pH regulation during increased fat processing. *Nat8* (N-acetyltransferase 8) and *Ces2c* (Carboxylesterase 2C) support detoxification of endogenous and exogenous compounds, protecting the liver from toxic insults, while down-regulation of *Fmo2/Fmo3* (Flavin-containing Monooxygenase 2/3), *Gucy2c* (Guanylate Cyclase 2C), *Odad4* (Outer Dynein Arm Assembly Factor 4) and *Apba1* (Amyloid Beta Precursor Protein Binding Family A Member 1) suggests balanced phase I metabolism. *Trhde* (Thyrotropin Releasing Hormone Degrading Enzyme), *Mtnr1a* (Melatonin Receptor 1A) and *Dio1* (Iodothyronine Deiodinase 1) mediate neuroendocrine and thyroid hormone regulation, adapting hepatic function to circadian and metabolic needs. *Erbb4* (Erb-B2 Receptor Tyrosine Kinase 4) and *Ntrk1* (Neurotrophic Tyrosine Kinase Receptor Type 1) are growth factor receptors supporting cell survival and anti-inflammatory/reparative signaling. *Gadd45a* (Growth Arrest and DNA-damage-inducible 45 alpha) and *Sgk2* (Serum/Glucocorticoid Regulated Kinase 2) integrate stress and cell cycle responses, supporting genomic stability and environmental adaptation. While the down-regulated genes reveal decreased requirements for rapid growth and remodeling, gene expression is increased to support robust metabolization of nutrients and readiness for detoxification. Comparing the GSEA gene list contributing to respiratory chain and oxidative phosphorylation with the GSEA list of genes responsible for fatty acid metabolism in 12-week-old mice underlines the organ-specific changes: while down-regulation of oxidative phosphorylation characterizes the heart, upregulation of fatty acid metabolism is unique to the transcriptional changes occurring in the liver (**Suppl. Figure 3**). Murine liver development has been extensively profiled by bulk and single-cell RNA sequencing, focusing on embryonal stages E12.5 to postnatal day 60 [14,15, Xu, 2022 #5519]. Our findings extend the time window from 8 to 12 weeks, demonstrating that the liver continues to shift to a metabolically active organ, centered on lipid handling and detoxification.

### Increased intestinal fatty acid metabolism

We next examined whether the transcriptional changes correspond to changes in murine metabolism between age 8 and 12 weeks. PCA of samples obtained from three different sites, intestine, plasma and urine, revealed age-dependent shifts (**Suppl. Figure 4**).

**Figure 4:**
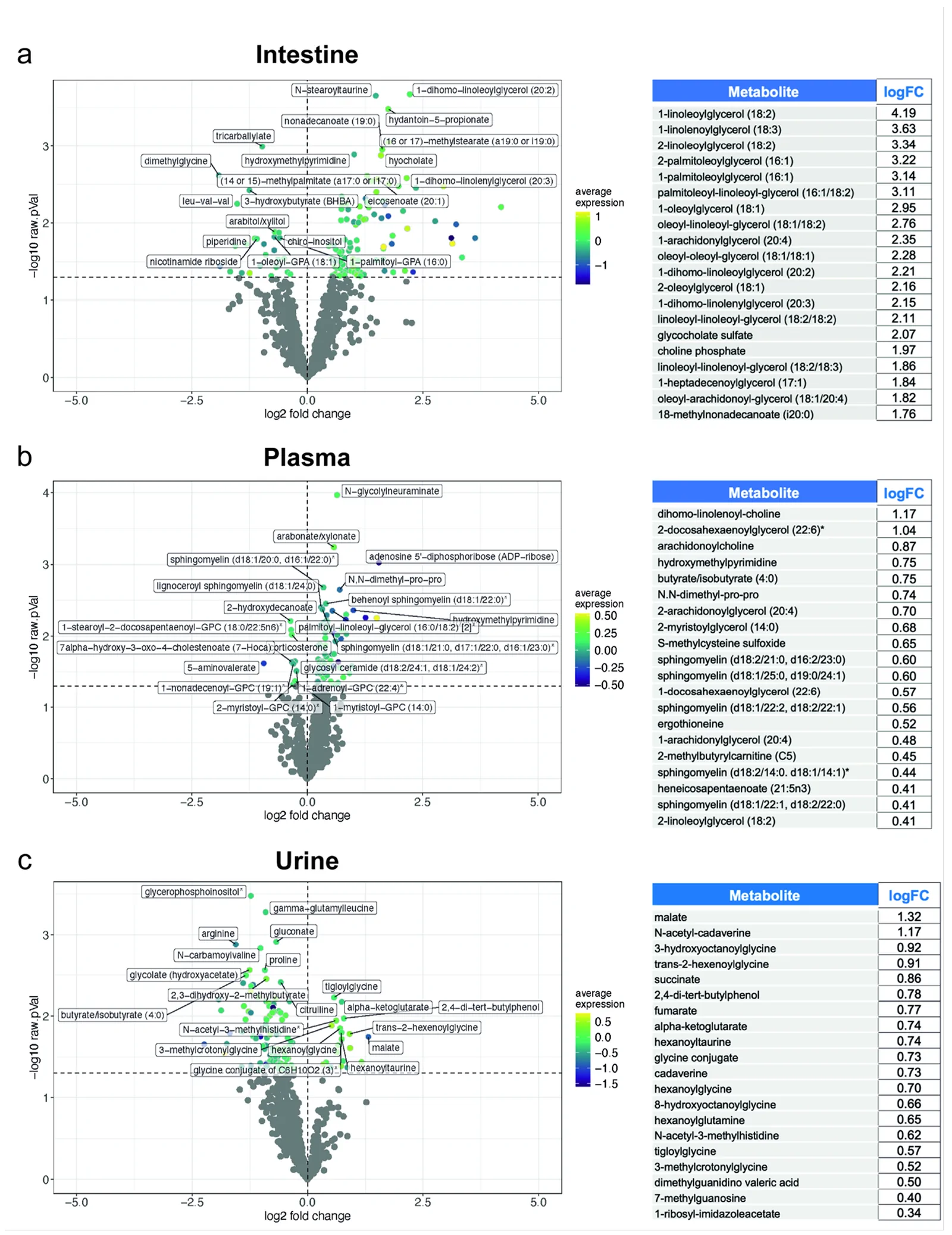
Age-dependent metabolic profiling identifies site-specific shifts in lipid handling and energy metabolism between 8 and 12 weeks of age. Volcano plots of metabolites identified in **(a)** intestine, **(b)** plasma, and **(c)**urine, accompanied by tables of top up-regulated metabolites (log2 fold change). Metabolomic analysis highlights systematic developmental changes: intestinal luminal samples show an increase in neutral lipid intermediates indicative of improved lipid absorption; plasma reveals an elevation in PUFA-rich glycerolipids and sphingomyelins reflecting membrane and lipoprotein remodeling; and urinary profiles demonstrate increased excretion of TCA cycle intermediates and acylglycines, confirming enhanced mitochondrial fatty acid β-oxidation and energy turnover during the transition to adulthood. Significant metabolites are depicted by colored dots, with the color scale indicating the average expression level across samples.

The intestinal samples showed a broad increase in neutral lipid intermediates, suggesting increased glycerolipid metabolism and re-esterification of fatty acids (Fig. 4A). The concurrent elevation of glycocholate sulfate and choline phosphate suggests additional perturbation of bile-acid handling and phospholipid metabolism, implicating altered lipid processing and possibly host–microbiota interactions in lipid homeostasis. The age-dependent increase in glycerolipid metabolites in the intestinal lumen from week 8 to week 12 in mice is likely driven by maturation of intestinal lipid handling and microbiota–host interactions. From weaning through early adulthood, the small intestine and cecum undergo functional maturation in lipid absorption, chylomicron assembly, and fatty-acid transporter expression [16]. As mice age from 8 to 12 weeks, improved uptake and processing of dietary triacylglycerols can lead to higher delivery of partially hydrolyzed glycerolipids into the intestinal lumen, explaining the observed age-dependent rise in these intermediates. Bile-acid composition and secretion mature post-weaning, increasing the detergent capacity for fat emulsification and hydrolysis by lipases [17]. This can enhance generation of monoacylglycerols and diacylglycerols from dietary triglycerides, which then appear proportionally higher in intestinal contents. Thus, the age-dependent increase in glycerolipid intermediates in the intestinal luminal content from weeks 8 to 12 likely reflects ongoing maturation of lipid absorption, bile-acid-mediated fat emulsification, and microbiota–host interactions.

### Alterations in plasma metabolites mirror intestinal fatty acid metabolism

The upregulation of the metabolites in murine plasma from 8 to 12 weeks corresponds to the age-dependent intestinal changes in lipid metabolism, with a shift toward omega-3-/polyunsaturated fatty acid (PUFA)-rich glycerolipids and sphingomyelins, changes in short-chain fatty-acid and amino-acid–related metabolites as well as altered endocannabinoid-related glycerolipid signaling (Fig. 4B).

The rise in dihomo-linolenoyl-choline and arachidonoylcholine indicates increased incorporation of PUFAs into choline-containing phospholipids, indicating age-related remodeling of membrane and lipoprotein lipids. The increase in butyrate and isobutyrate suggests enhanced microbial production of short-chain fatty acids (SCFAs) and their subsequent entry into the systemic circulation, consistent with age-dependent maturation of the gut microbiota and colonic fermentation capacity. These changes were concordant with the targeted SCFA analysis (**Suppl. Figure 5**).

The increase in N,N-dimethyl-pro-pro and 2-methylbutyryl-carnitine (C5) hints at altered branched-chain amino-acid (BCAA) metabolism and mitochondrial β-oxidation, often seen during growth and increasing energy demand [18]. Ergothioneine and S-methylcysteine sulfoxide are sulfur-containing compounds with antioxidant and cytoprotective functions [19, 20]. Their upregulation may reflect increasing systemic demand for redox buffering as mice grow and encounter greater oxidative and inflammatory stimuli. Collectively, these alterations suggest an age-dependent maturation of lipid and choline metabolism, with enhanced microbial fermentation and membrane-lipid remodeling.

### Liver-Derived Ketogenesis Enhances Metabolic Efficiency

Increased expression of liver genes involved in fatty acid metabolism, phospholipid remodeling, ketogenesis, and sphingolipid biosynthesis are likely responsible for the increase of corresponding plasma metabolites [21, 22]. *Acsl1* (Acyl-CoA Synthetase Long-chain 1) activates long-chain fatty acids for both β-oxidation and lipid synthesis, increasing phospholipid, choline and sphingomyelin metabolites, while the Acyl-CoA thioesterases (*Acot2*, *Acot8*, *Acot12*) increase acylglycerols and lipid intermediates (**Suppl. Table 2**).

Of note is the increased mitochondrial production of ketone bodies (3-hydroxybutyroylglycine, 2S,3R-dihydroxybutyrate) and short- and medium chain fatty acids (butyrate/isobutyrate (4:0), 2-methylbutyrylcarnitine (C5), cis-4-decenoate (10:1n6)), likely linked to increased expression of *Acadm* (acyl-CoA Dehydrogenase, medium chain) and *Hadha/Hadhnb* (Hydroxyacyl-CoA Dehydrogenase subunits) (**Suppl. Figure 6**). Ketone body production is supported by peroxisomal breakdown of fatty acids, involving *Ehhadh* (enoyl-CoA hydratase/3-hydroxyacyl-CoA dehydrogenase) and *Acaa1a/Acaa2* activity (acetyl-CoA acyltransferases) [23] (**Suppl. Figure 6**). Together these two pathways drive the conversion of fatty acids to ketone bodies and their derivatives in the liver, resulting in increased delivery of these alternative energy substrates to blood and peripheral tissues, including the heart [24]. Per molecule of oxygen consume, the ATP yield is higher for ketone body oxidation than for fatty acid oxidation. Thus, less oxidative capacity is required to generate the same ATP production and cardiac output, providing a possible explanation for decreased gene expression associated with mitogenesis and oxidative phosphorylation. The liver typically increases ketone production during states of low insulin or high glucagon concentrations, which are caused by fasting, prolonged exercise, low-carbohydrate diets and starvation [22]. In late adolescent mice, increased ketogenesis may result from a combination of enhanced lipolysis due to hormonal shifts and elevated growth hormone causing transient insulin resistance [25].

### Renal maturation in response to systemic metabolic reprogramming during early adulthood

The upregulation of the urinary metabolites in mice from 8 to 12 weeks of age points to two primary developmental processes: increased fatty acid oxidation (FAO) flux and ongoing maturation of metabolic turnover in the liver and kidney (Fig. 4C). The elevation of various acylglycines strongly indicates increased flux through mitochondrial β-oxidation. These acylglycines are formed when surplus acyl-CoA intermediates are conjugated with glycine for excretion, a hallmark of shifting energy substrate reliance during early adulthood [26]. The increase in malate, succinate, fumarate, and α-ketoglutarate suggests enhanced mitochondrial respiration and anaplerotic activity, likely reflecting the higher metabolic demands and energy turnover typical of this active growth phase. The presence of cadaverine and N-acetyl-cadaverine in the urine is frequently linked to gut-microbial decarboxylation of lysine and subsequent host-mediated processing [27]. Their rise suggests progressive expansion and/or shift in the gut microbiota, leading to increased systemic availability of these amines as the mouse matures.

Collectively, the metabolic changes point to a coherent developmental transition during late mouse adolescence/early adulthood with the gut exerting a robust increase in hydrolysis of triglycerides. These products appear to be absorbed and reprocessed into circulating PUFA-rich lipids and sphingomyelins, which are reflected in the plasma as maturing lipoprotein and membrane components. The urinary excretion of TCA cycle intermediates and acylglycines is a clear hallmark of increased mitochondrial energy turnover and fatty acid β-oxidation, consistent with the heightened metabolic demands of a growing mouse.

### Changes in the gut microbiome drive metabolic shifts during adolescence

**Figure 5:**
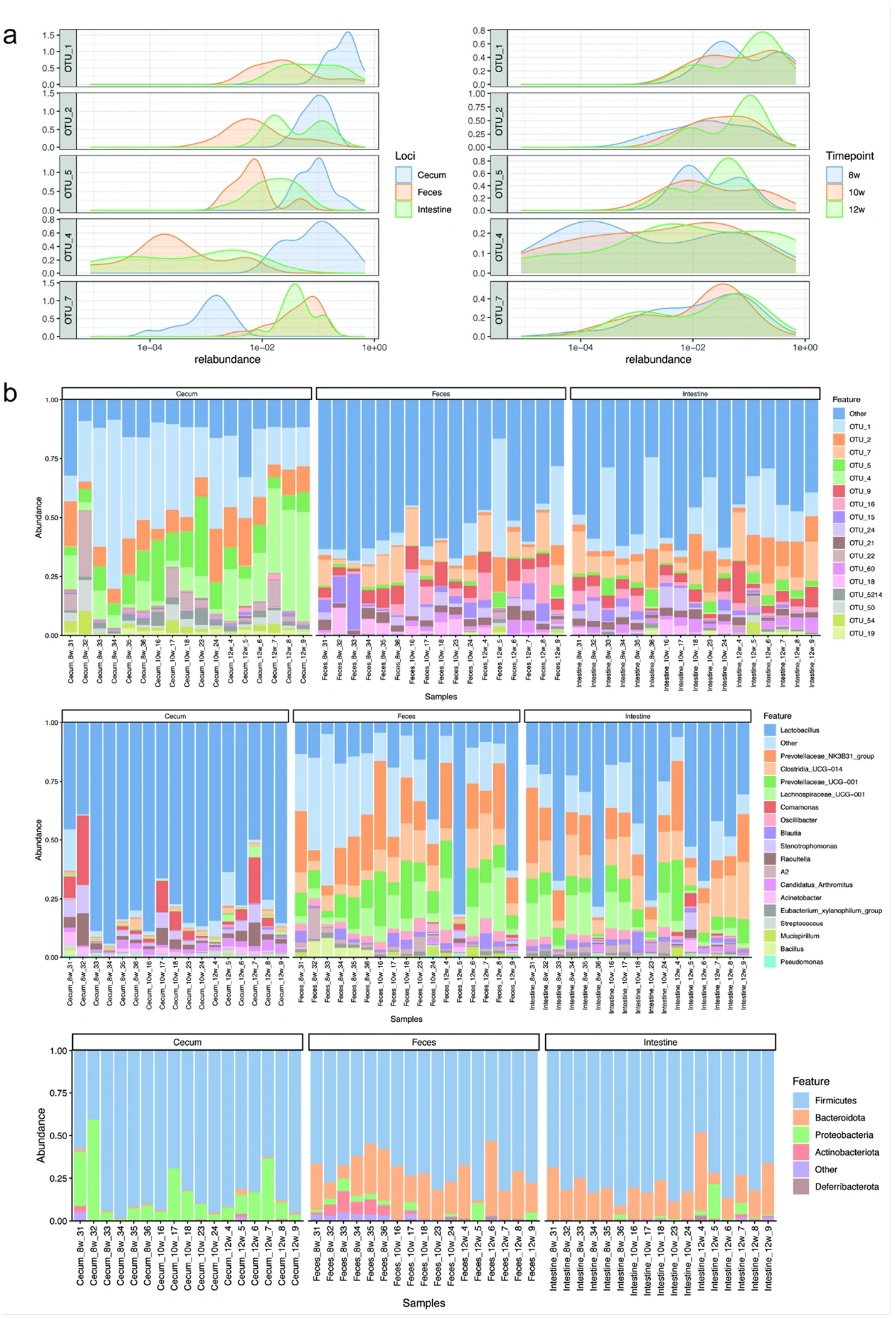
Site-specific dynamic remodeling of the microbiome during the 8- to 12-week transition. **(a)** Dynamic remodeling of dominant microbial taxa across anatomical sites and timepoints. Density plots show log10-scaled relative abundance distributions for the five most abundant taxonomic features: Lactobacillus johnsonii, Lactobacillus murinus, Lactobacillus reuteri, Cyanobacteriia, and Bacteroidia. Distributions are shown separately by anatomical site, including cecum, feces, and small intestine, and by age, comparing 8-, 10-, and 12-week samples. Curves indicate the density of samples at each relative abundance level, revealing shifts in microbial community composition across intestinal locations and during the 8- to 12-week transition. **(b)** Taxonomic composition of the microbiome of samples from the cecum, feces, and small intestine. Stacked bar chart illustrating the relative abundance of micro bial features (OTUs) across individual mouse samples. Samples are labeled by cage number followed by the sampling time point: 8 weeks (2d), 10 weeks (2w), and 12 weeks (4w). Each bar represents the total community composition of a single sample, with color-coded segments indicating the proportion of each OTU. The other category encompasses low-abundance taxa, highlighting the inter-individual and longitudinal shifts in community structure within the small intestine.

The comparison between the microbiome of the small intestine and the cecum displayed compartmentspecific microbiome shifts with some shared age-related signals between the small intestine and cecum (Fig. 5, **Suppl. Figure 7**).

The small intestine showed a broad set of changes, with several taxa shifting clearly at 12 weeks relative to 8 weeks of age. Prominent increases appeared for taxa such as *Pseudomonas*, *Streptococcus*, *Flavonifractor*, *Candidatus saccharimonas*, *Parvibacter*, and *Mucispirillum*. A few taxa showed decreases or weak/near-null effects, including *Agathobacter* and some lower abundant groups.

The cecum showed a somewhat more conserved but still clearly shifted pattern that was not identical to the small-intestinal pattern, indicating that the small intestine and cecum were not simply mirroring each other. At 10 and 12 weeks, taxa such as *Agathobacter*, *Sphingomonas*, *Hafnia-Obesumbacterium*, *Megasphaera*, *Bifidobacterium*, *Bosea*, and *Blautia* were generally shifted upward relative to 8 weeks, while *Psychrobacter* and *Methylobacterium-Methylorubrum* trended downward. *Bifidobacterium* can generate lactate and cross-feed *Megasphaera* to promote butyrate formation [28], while *Agathobacter* and *Blautia* are commonly linked to carbohydrate fermentation and butyrate-related ecology [29]. The increase in *Sphingomonas*, *Hafnia-Obesumbacterium*, and *Bosea* likely reflect broader ecological remodeling rather than enrichment of canonical gut commensals, since *Sphingomonas* and *Bosea* are predominantly environmental taxa, whereas *Hafnia* species can occur in the intestinal flora but are not established as core beneficial microbes [30].

The small intestine showed more proximal, potentially exposure-sensitive taxa, whereas the cecum revealed a different set of taxa that may reflect more stable fermentation-associated communities, consistent with increased fermentation output and altered bile-acid metabolism as well as PUFA-containing glycerolipids (Fiugre 6). In particular, *Clostridia* and *Lachnospiraceae* (like *Blautia*) are producers of short-chain fatty acids (SCFAs) through the fermentation of dietary fibers, contributing to the increase in butyrate (HMDB0000039) (logFC = 0.746) [31]. The lipid-signaling molecules endocannabinoids 2-arachidonoylglycerol (logFC = 0.702) and 2-docosahexaenoylglycerol were also increased (logFC = 1.041), suggesting that the gut microbiota is actively modulating endocannabinoid homeostasis, which in turn influences inflammation and energy homeostasis [32, 33].

Collectively, these changes suggest that the gut microbiome at the 12-week timepoint becomes metabolically more active regarding fiber fermentation compared to the 8-week baseline. The systemic rise in butyrate is a positive indicator for gut epithelial health and barrier integrity as well as enhancing the metabolic pool of beneficial fatty acids in the blood.

### Conclusion

Comprehensive multi-omics analysis demonstrates that male C57BL/6 mice between 8 and 12 weeks of age undergo pronounced, organ-specific transcriptional and metabolic maturation. During this period, the heart transitions to a maintenance phenotype characterized by reduced oxidative phosphorylation and enhanced structural and contractile development. The kidney develops immune-active features and refines its transport and homeostatic functions. Concurrently, the liver becomes central to lipid metabolism, detoxification, and ketogenesis, as indicated by upregulation of fatty-acid metabolic pathways and ketone body formation, with a decline in extracellular matrix-related growth signatures. Metabolomic profiling of intestinal, plasma, and urinary samples further supports increased fatty-acid processing, elevated production of microbiome-derived short-chain fatty acids, and enhanced hepatic ketogenesis. Collectively, these findings highlight that the interval from 8 to 12 weeks marks an ongoing phase of coordinated developmental remodeling across major organs and systemic metabolism, reflecting the physiological transition from late adolescence to early adulthood in mice.

## Material and methods

### Animals experiments

All animal experiments were approved by the local authorities (Regierungspräsidium Freiburg, 35-9185.81/G-22/015) and were conducted in accordance with German animal welfare regulations. Six-week-old healthy male C57BL/6NCrl mice were purchased from Charles River Laboratories and randomly assigned to three groups based on age at sacrifice: an 8-week group (n = 6), a 10-week group (n = 5), and a 12-week group (n = 6). Mice were housed at 2–3 animals per cage to reduce the effects of coprophagy on gut microbiota composition. Animals were maintained under standard housing conditions with a 12-hour light/dark cycle and had ad libitum access to food and water. Environmental enrichment (paper nesting material) was provided. Body weight, food intake, and water consumption were measured twice weekly. Mice were fed a standard rodent chow diet (ssniff Spezialdiäten GmbH, Germany).

### Sample collection

Urine and feces were collected prior to anesthesia. Mice were then anesthetized via intraperitoneal injection of ketamine/xylazine (120 and 8 μg/g body weight, respectively), Blood samples were collected via retro-orbital sinus bleeding. Plasma was obtained by centrifugation at 2,500 × g for 10 min at 4°C. Subsequently, mice were euthanized by cervical dislocation. Kidney, liver, and heart tissues, as well as small intestinal and cecal contents, were collected. Intestinal contents were obtained by gently extruding the intestine with sterile forceps. All samples were immediately flash-frozen in liquid nitrogen and stored at − 80°C until further processing.

### Transcriptomic data preprocessing and analysis

Mouse kidney, liver, and heart tissues collected from mice at 8, 10, or 12 weeks of age were homogenized on ice in lysis buffer (RLT buffer) from the RNeasy Mini Kit (Qiagen, Hilden, Germany; Cat. No. 74104) using an IKA^®^ T10 basic homogenizer at maximum speed for 30 s. Total RNA was then extracted according to the manufacturer’s instructions. RNA quality and integrity were assessed using a NanoDrop^™^ spectrophotometer and the Agilent 2100 Bioanalyzer system. High-quality RNA samples were sent to Novogene Bioinformatics Technology Co., Ltd. for library preparation and sequencing. Library construction was performed according to standard protocols, and sequencing was carried out on an Illumina NovaSeq platform to generate approximately 30 million paired-end reads (2 × 150 bp) per sample.

Transcriptomic data were generated from heart, kidney, and liver, with 12 samples per tissue, comprising four biological replicates from each age group: 8, 10, and 12 weeks. Read quality was assessed using *FastQC* v0.11.9 (http://www.bioinformatics.babraham.ac.uk/projects/fastqc). Raw reads were processed with *cutadapt* v4.2 [34] to remove adapters, poly-G artifacts, and low-quality bases. Reads were discarded if they contained more than two expected errors, more than 30% ambiguous bases, or were shorter than 20 nucleotides after trimming. Processed reads were aligned to the mouse reference genome GRCm39 from *Ensembl* v113 [Yates *et al.*, 2026] using *STAR* v2.7.11b [35], and gene-level counts were generated with --*quantMode GeneCounts*.Downstream analyses were performed in R separately for each tissue. Genes were retained if, in at least one age group, they had non-zero raw counts in at least two samples and an average raw count of at least 10. Principal component analysis was performed using centered log2CPM-transformed expression values. Differential gene expression analysis was conducted using *edgeR* v4.8.2 [36] and *limma* v3.66.0 [37]. Library sizes were normalized using the trimmed mean of M-values method implemented in *edgeR::calcNormFactors*. Age was modeled using the design ~ *0 + age*. Genes with adjusted *p*-value < 0.05 were considered differentially expressed. For gene set enrichment analysis, mouse gene sets were obtained directly from MSigDB using the R package *msigdbr* v26.1.0 (https://igordot.github.io/msigdbr/). For each tissue and contrast, genes were ranked by moderated t-statistic, and enrichment analysis was performed using the GSEA function with the fgsea method in *clusterProfiler* v4.18.4 [38]. Gene sets with adjusted *p*-value < 0.05 were considered significantly enriched. For pathway visualization, KEGG oxidative phosphorylation genes were matched to differential expression results for each tissue and contrast. Entrez identifiers were converted to mouse KEGG format to generate KEGG Mapper input files [39].

### Untargeted global metabolites analysis

Urine, plasma, and cecal content samples collected from mice at 8, 10, or 12 weeks of age were submitted to Metabolon Inc. (Morrisville, NC, USA) for untargeted global metabolomic profiling. Samples were prepared using the automated MicroLab STAR^®^ system (Hamilton Company). Recovery standards were added prior to the first extraction step for quality control. Proteins were precipitated with methanol under vigorous shaking for 2 min using a GenoGrinder 2000, followed by centrifugation to remove proteins, dissociate small molecules bound to protein, and recover chemically diverse metabolites. The resulting extract was divided into five fractions: two for analysis by RP/UPLC-MS/MS with positive ion mode ESI, one for RP/UPLC-MS/MS with negative ion mode ESI, one for HILIC/UPLC-MS/MS with negative ion mode ESI, and one reserved as backup. Samples were briefly placed on a TurboVap^®^ to remove organic solvent and stored overnight under nitrogen before analysis.

Quality control procedures included pooled matrix samples (used as technical replicates across the dataset), process blanks (extracted water), and a cocktail of non-interfering QC standards spiked into each sample to monitor instrument performance and support chromatographic alignment. Instrument variability was assessed as the median relative standard deviation (RSD) of QC standards added prior to injection into the mass spectrometer. Overall process variability was determined as the median RSD of endogenous metabolites consistently detected in pooled matrix samples. Experimental samples were randomized across the analytical run, with QC samples interspersed at regular intervals. Metabolomic profiling was performed using a Waters ACQUITY ultra-performance liquid chromatography system coupled to a Thermo Scientific Q-Exactive high-resolution accurate mass spectrometer equipped with a heated electrospray ionization source and Orbitrap mass analyzer operated at a resolution of 35,000. Dried extracts were reconstituted in method-specific solvents containing standards at fixed concentrations to ensure consistent injection and chromatography. Four complementary LC-MS/MS methods were applied, including two acidic positive ion methods optimized for hydrophilic and hydrophobic compounds, a basic negative ion method using a dedicated C18 column, and a negative ion HILIC method. Mass spectra were collected over 70–1000 m/z with alternating MS and data-dependent MS/MS scans using dynamic exclusion. Raw data were processed with the standard metabolomic workflow.

For each sample type, 12 samples were analyzed, comprising four biological replicates per age group. Data preprocessing followed Metabolon’s sample-type-specific normalization recommendations. Unnormalized peak area data, reported as total ion counts, were used as input. Raw values were median-scaled, and missing values were imputed separately for each metabolite using the minimum observed value for that metabolite. Sample-type-specific normalization was then applied: cecal metabolites were normalized to total cecal content, plasma metabolites to extracted plasma volume, and urine metabolites to targeted creatinine abundance. Normalized intensities were log-transformed and used for downstream analyses.

Principal component analysis was performed separately for cecal content, plasma, and urine using log-transformed normalized metabolite intensity matrices. Differential metabolite abundance analysis was performed in R using *limma* v3.66.0 [40] separately for each sample type. Age group was modeled using the design *~ 0 + age*. Metabolites with raw *p*-value < 0.05 were considered differentially abundant.

### Short chain fatty acid (SCFA) analysis

Cecal content samples collected from mice at 8, 10, or 12 weeks of age were analyzed for short-chain fatty acids (SCFAs). Samples were submitted to Metabolon Inc. (Morrisville, NC, USA) for targeted SCFA quantification. SCFAs, including acetic acid (C2), propionic acid (C3), isobutyric acid (C4), butyric acid (C4), 2-methylbutyric acid (C5), isovaleric acid (C5), valeric acid (C5), and caproic acid (hexanoic acid, C6), were quantified using liquid chromatography–tandem mass spectrometry (LC–MS/MS) on an Agilent 1290 UHPLC system coupled to a SCIEX QTRAP 5500 mass spectrometer. Plasma and cecal content samples were spiked with stable isotope-labeled internal standards, homogenized, and subjected to protein precipitation using an organic solvent. After centrifugation, the supernatant was collected and derivatized. The derivatized samples were diluted and injected into an Agilent 1290 UHPLC/AB Sciex QTrap 5500 LC-MS/MS system equipped with a C18 reversed-phase column. The mass spectrometer was operated in negative electrospray ionization (ESI) mode. Peak areas of analyte-specific product ions were quantified relative to corresponding internal standards. Quantification was performed using weighted linear least-squares regression based on calibration standards prepared prior to each analytical run. Raw LC-MS/MS data were acquired using Analyst software version 1.6.2 (AB SCIEX) and processed using SCIEX OS-MQ software version 1.7. Data reduction was performed using Microsoft Excel for Office 365 (v16).

### 16S rRNA microbiome data preprocessing and analysis

Small intestinal contents, cecal contents, and feces were collected from mice at 8, 10, or 12 weeks of age and analyzed using 16S rRNA gene sequencing.

Briefly, Bacterial genomic DNA was extracted from approximately 100 mg of each sample using the TIANamp Stool DNA Kit (TIANGEN, Beijing, China; Cat. No. DP328-02) according to the manufacturer’s instructions. The V4 region of the bacterial 16S rRNA gene was amplified by PCR using the universal primer pair 515F–806R (forward: 5′-GTGYCAGCMGCCGCGGTAA-3′; reverse: 5′-GGACTACNVGGGTWTCTAAT-3′) [41]. PCR products of the expected size were verified and purified using 2% agarose gel electrophoresis. Purified amplicons from each sample were quantified, and equal amounts were pooled. The pooled DNA was subjected to end repair, A-tailing, and ligation with Illumina sequencing adapters to construct sequencing libraries. The libraries were sequenced on an Illumina platform using a 250 bp paired-end strategy (Illumina PE250), with an average sequencing depth of approximately 100,000 raw reads per sample. DNA extraction, library preparation, and sequencing were performed by Novogene (Novogene Bioinformatics Technology Co., Ltd., which provided an OTU (Operational Taxonomic Unit) count table as a feature-by-sample matrix with taxonomic annotations in the final column. Taxonomic annotations were extracted and mapped to the corresponding OTUs. Microbiome profiles were generated from cecal contents, fecal samples, and small intestinal contents, with 17 samples per site, including six 8-week-old, five 10-week-old, and six 12-week-old mice. Preprocessing and downstream analyses were performed in R using the *mia* package v1.19.2 (https://microbiome.github.io/mia/), based on the *TreeSummarizedExperiment* framework [42]. Relative abundances were calculated for quality control and exploratory assessment of library sizes, count and abundance distributions, and feature density across samples. Low-information features were removed by excluding OTUs with missing genus-level annotations, zero variance across samples, or low prevalence. OTUs annotated as Mitochondria were also excluded, as these sequences are likely derived from host or dietary sources rather than microbial taxa of interest. For prevalence filtering, OTUs were considered detected at ≥ 1 count and retained if present in at least 10% of samples. Relative abundances were recalculated after filtering. Community composition was assessed using filtered relative abundances. The most abundant microbes were identified separately for cecum, feces, and small intestine samples; their union was retained for visualization, while remaining taxa were grouped as “Other”. Alpha diversity was calculated at the OTU level using *addAlpha* in *mia* v1.19.2 (https://microbiome.github.io/mia/) on raw counts with a detection threshold of 1. Observed richness, Berger-Parker dominance, and Shannon diversity were used to quantify taxon richness, dominance, and evenness, respectively, and visualized as boxplots across age groups and sampling sites. Differential abundance analysis was performed at the genus level separately for each anatomical site using *maaslin3* v1.2.0 [43]. Genus-level abundance was modeled as a function of age group. The *maaslin3* workflow converted counts to relative abundances, replaced zeros with half of the smallest non-zero relative abundance per taxon, applied log2 transformation, and performed linear modeling to identify taxa associated with age-dependent microbiome changes.

## Supplementary Material

This is a list of supplementary files associated with this preprint. Click to download.

- Suppl.Table1GSEAheartage12wage8w.xlsx
- Suppl.Table2GSEAkidneyage12wage8w.xlsx
- Suppl.Table3GSEAliverage12wage8w.xlsx
- Suppl.Table4DGEheartage12wage8w.xlsx
- Suppl.Table5DGEkidneyage12wage8w.xlsx
- Suppl.Table6DGEliverage12wage8w.xlsx
- SupplementalInformation.pdf

## Figures and Tables

**Figure 6:**
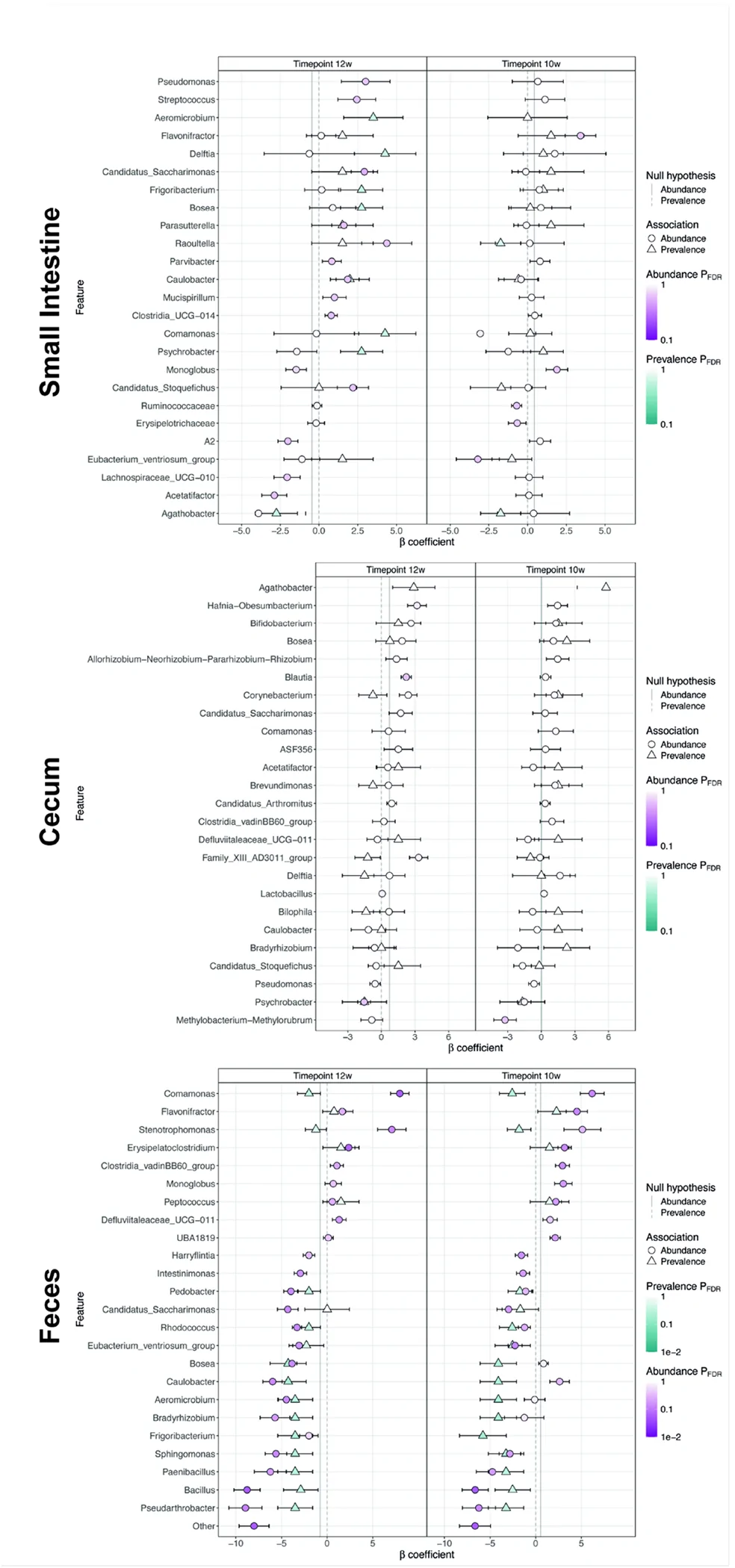
Compositional changes in the gut microbiome during late adolescence (10 vs. 12 weeks). Forest plots showing beta-coefficients for microbial features in the small intestine and cecum. Results represent associations with microbial abundance (circles) and prevalence (triangles) across time points. Significant findings are indicated by color-coded values (purple for abundance, teal for prevalence), highlighting the microbial shifts that accompany the transition from 10 to 12 weeks of age. Error bars represent 95% confidence intervals, and the vertical dashed line at 0 denotes the null hypothesis.

## Data Availability

The RNA sequencing data is available at the NCBI Gene Expression Omnibus (GEO) (accession number: GSE337595. The 16S rRNA microbiome data is available in the NCBI Sequence Read Archive (accession number: PRJNA1491087). The metabolomics data is available at the NIH Common Fund’s National Metabolomics Data Repository (NMDR) website, the Metabolomics Workbench, https://www.metabolomicsworkbench.org where it has been assigned Project ID PR003206 and Project DOI https://doi.org/10.21228/M88P1N. [44].
